# Modification of the vacuum-steam thawing method of meat by using the initial stage of sublimation dehydration

**DOI:** 10.1038/s41598-022-12114-7

**Published:** 2022-05-12

**Authors:** Adam Kopeć, Sylwia Mierzejewska, Aldona Bać, Jarosław Diakun, Joanna Piepiórka-Stepuk

**Affiliations:** Division of Food Industry Processes and Facilities, Department of Mechanical Engineering, Koszalin University of Technology, Racławicka Street 15-17, 75-620 Koszalin, Poland

**Keywords:** Engineering, Thermodynamics

## Abstract

Vacuum-steam thawing is one of the methods used for defrosting food, realized in the atmosphere of water vapour under the conditions of reduced pressure. The water vapour formed in vacuum with the temperature of 20 °C fills the defrosting chamber and condenses on the surface of the defrosted product. The condensated steam has the role of thermal energy carried enabling product thawing. The study presents a modification of this method, introducing an additional stage of sublimation-dehydration vacuum steam thawing (SRVST). The study was carried out for different variants of initial sublimation degree (in the range from 0 to 15%) of a slice of pork loin (m. longissimus lumborum) assessing the final effect of the process of vacuum-steam thawing. Thawing kinetics was determined with the SRVST method, degree of sample defrosting and level of their rehydration. Based on the results it was demonstrated that the use of 12% sublimation dehydration of a meat sample enables its complete defrosting (reaching the temperature not exceeding the cryoscopic temperature).

## Introduction

Considering that frozen foods, in particular meat and fish, are used in food processing, the thawing operation is necessary. Along with freezing and storage, this process has a significant impact on the quality of food products. It should be conducted conditions ensuring the most complete restoration of the original characters of the product. To this end, overheating of the product surface during its thawing and defrosting drip loss should be limited, which are causes of significant quality losses and physical, biochemical and microbiological changes of the product^1–6^. Numerous thawing methods are available^7–12^. The most well known methods of meat raw materials thawing are traditional methods—air thawing and water or brine immersion thawing. This thawing method is rather prolonged (it may last for up to 3 days), requires ensuring large storage surface area and it forms a significant microbial hazard due to the possibility of cross contamination^13–15^. The defrost chambers for contact or steam–air defrosting are an alternative to this method. In the recent years, a continuous interest has been expressed in the study on finding new defrosting technologies, such as the use of high pressure microwave, ultrasonic and infrared^8,12,14,16,17^. Research on dielectric, radio wave and high-voltage electrostatic field thawing has been conducted^5,9,18–20^. A series of studies concerns modifications of known methods, i.e. vacuum-steam, sublimation contact or sublimation-vacuum-steam thawing^10,11,17,21,22^. The latter method is covered by the present study.

Following the methodology of steam thawing, the thermal energy is provided by means of water vapour condensation on the surface of the defrosted food products. This heat, required for the phase change of ice into water in a frozen raw material is obtained from the phase change from steam to water. The steam thawing variant consists in the application of underpressure for the process, known as vacuum-steam thawing (VST). The product defrosted using this method is place in a vacuum chamber, from which air is pumped away. An open vessel with water is located on the bottom of the chamber, or the chamber is linked to an external tank. As a result of this underpressure (approx. 2400 Pa), water starts boiling at room temperature (approx. 20 °C). In order to maintain the water in the boiling state it must be heated. This is normally done by heating with steam, sometimes through a water heat exchanger or electric heaters. The condensation heat is used by the frozen product, resulting in its quick defrosting (approx. 120 g of condensing water defrosts 2 kg of frozen product)^22,23^. A favorable outcome of this method is the fact that thawing with vacuum steam takes place at the temperature of 20 °C. In this manner, overheating of the surface of defrosted food products is avoided, which takes place in the case of defrosting in normal pressure with steam with the temperature of 100 °C^19,23–27^. In the 1970s industrial solutions for vacuum-steam thawing were executed. However, this technology was withdrawn due to the negative phenomenon of significant drip loss. Authors have modified this method of vacuum-steam defrosting by introducing an additional stage of preliminary dehydration of the material. It was assumed that during ice sublimation from products with cellular structure, ice would sublimate in an easier manner from the channels and intercellular space than from the inside of cells. Thus, an inner porous structure is formed^11,20,25,28,29^. The modification was named sublimation-dehydration vacuum steam thawing (SRVST).

Water accounts for about 75% of lean pork meat^1,30^. Different process operations, i.e. cutting, heating, grinding, pressing and in particular freezing–thawing of meat result in numerous unfavorable qualitative changes that depend on the level of weight loss. Under optimum conditions for freezing, storage and thawing of pork, the weight losses related to the defrosting drip loss reach 10%, while in the case of deviations they can reach even 18%^31^. This is caused by the brief defrosting time and absence of rehydration of the thawed aqueous phase. Storage of meat at a temperature of − 20 °C results in freezing about of the 80% water it contains^19,24,32^, then 60% of frozen meat is ice (75% water content × 80% frozen water = 60% ice in the meat). According to Eq. (1), the heat (Q_t_) needed for meat defrosting (m = 0.6 · m_m_) is a product of the mass of meat (m_m_) and mass fraction quantity ice contained it (0.6) multiplied by the heat of ice-water phase change (c_t_ = 335 kJ/kg).

1 $$ {\text{Q}}_{{\text{t}}} = 0.{6} \cdot {\text{m}}_{{\text{m}}} \cdot {\text{c}}_{{\text{t}}} \,\left[ {{\text{kJ}}} \right] $$

The heat provided to the product by the condensing steam, that is the heat of the steam—liquid phase change is c_p_ = 2260 kJ/kg. Thus, the amount of steam (m_p_) needed to defrost a certain mass of meat was determined by Eq. (2):

2 $$ m_{p} = \frac{{Q_{t} }}{{c_{p} }} = \frac{{0.6 \cdot m_{m} \cdot c_{t} }}{{c_{p} }} $$

For the check, the weight of the steam required to defrost (m_p_) in relation to the weight of the meat (m_m_) is:

$$ \frac{{m_{p} }}{{m_{m} }} = \frac{{0.6 \cdot c_{t} }}{{c_{p} }} = \frac{0.6 \cdot 335}{{2260}} = 0.09 $$

When these data are taken into account in the heat balance, it is demonstrated that when 9% of water mass is sublimated from frozen meat and forming water vapour is introduced in its place (the porous structure), the material will be entirely defrosted.

Based on the above circumstances a hypothesis was drawn up stating that if approximately 9% of ice is sublimated from frozen meat, then after the introduction of steam in thus created porous structure of meat, it will be defrosted in its entire volume with the bypassing of heat penetration from the surface. During vacuum-steam defrosting steam condenses on the surface of the defrosted material, and the heat penetrates the material based on the conductivity principle. However, the application of sublimation dehydration in the initial stage of vacuum-steam defrosting will result in the penetration (absorption) of water vapour into the porous structure of the defrosted material (at the stage of chamber steaming) and its condensation within the material. The condensing steam transfers the heat from the condensation phase change, resulting in even thawing of the material throughout its volume, thus limiting surface defrosting and defrosting drip loss.

The aim of the study was to demonstrate the possibility of sublimation application as a preliminary stage of meat tissue defrosting with vacuum-steam method and determination of the effect of water sublimation on the level of defrosting of a meat portion. Based on the hypothesis made, application of sublimation would result in formation of a porous structure of the defrosted material, which would enable steam absorption and thus shorten the defrosting process. Based on the research, the influence of the degree of water sublimation on the thawing level of meat sample and the degree of rehydration were determined as well as the characters of these interactions. The intermediate aim of the work was to reduce the time thawing of meat sample while maintaining the quality of the raw material, determined by the level of initial weight recovery—level of rehydration (S_r_).

## Materials and methods

### Research material

The research material consists of the *longissimus dorsi muscle* of a male fattening pig of the Polish Landrace breed (meat type) with a weight of 100 kg, collected from the lumbar part (*m. longissimus lumborum*). The carcass was stored in a coldstore for two days after the slaughter. Meat sample was prepared by crosswise cutting of the muscle fibers to a thickness of approx. 20 mm and weight 100 g ± 5 g (length approx. 70 mm, width approx. 30 mm). In this manner, 21 samples of meat were prepared (3 repetitions for each sublimation variant). The samples with thermocouple tip inserted into them^6^ were convection frozen to a temperature of − 30 °C and were stored at this temperature for a period of 2 weeks. After this time the samples were defrosted using sublimation-dehydration vacuum steam thawing.

### Research stand

The vacuum chamber consisted of a cylindrical vessel (1) covered with a glass plate (2) enabling observation of the process, connected with a vacuum pump (3). The chamber steaming was realized using the steam obtained from a heated water tank (4) connected with the chamber (1) via a duct with valve. The frozen meat sample (5) was placed in a vacuum chamber under a weight sensor (7). The measurement signals from the thermocouple (6) (type K thermocouple, rod thickness 0.2 mm, measurement error ± 1 °C) and weight sensor (7) (scale module type IL 0.2, by Mensor, accuracy class III, verification scale e = 0.01 g) collected by an analog–digital card (8) transferred to a computer and processed using MatLAB software. The measured values (3 replications for each variant) were archived in the computer memory. During sublimation two IR lamps (9) with 2 × 5 W were switched on, providing heat to the sublimated material (5).

**Figure 1:**
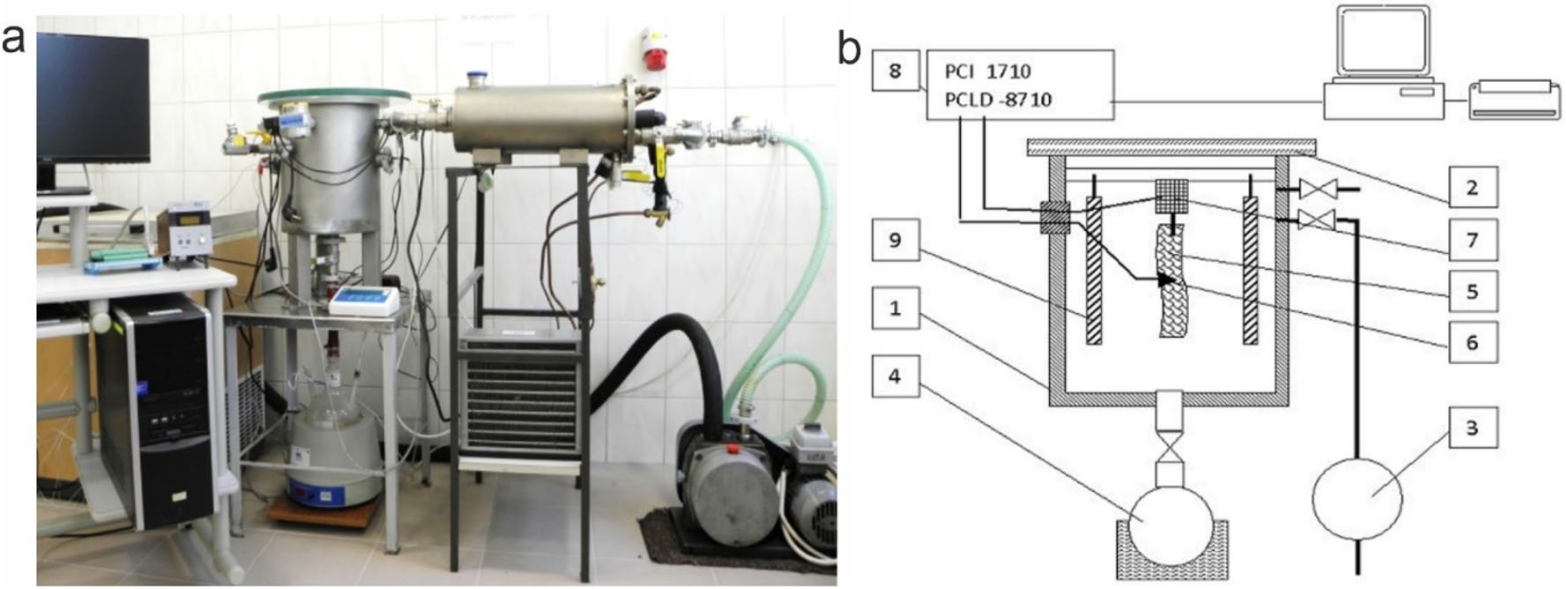
Test stand of sublimation vacuum steam thawing: (**a**) Layout of the equipment; (**b**) Diagram of the experimental setup: 1—vacuum chamber container, 2—glass cover, 3—vacuum pump, 4—water tank with a heater, 5—meat sample, 6—K-type thermocouple wire (NiCr-NiAl), 7—IL 0,2-type weight modulus manufactured by Mensor, 8—PCLD-8710-type plate connected with a PCLD-8710-type temperature compensator and a PCI-1710-type measuring card, 9—infrared radiators.

View of the experimental system and diagram with measurement elements significant for the contents of the present paper are presented in Fig. 1a,b.

### Thawing process

A frozen meat sample with thermocouple was suspended on the weight measurement sensor. The thermocouple and the weight sensor were connected to the measurement chain and then the chamber was closed with the cover. The vacuum-steam defrosting process was preceded by the preliminary stage of meat dehydration. To this end, the vacuum pump was started with water tank valve closed. After vacuum at the level of 50 Pa was obtained, IR lamps were switched on and sublimation process was conducted until the assumed dehydration level was obtained (S_s_ [%] = 0; 2; 5; 8; 10; 12; 15). At the same time, the sample weight loss due to its sublimation dehydration was monitored. After obtaining the appropriate sample weight, the IR lamps were switched off, the vacuum pump suction valve was closed and the valve to the water tank was opened, resulting in chamber steaming. As a result of heating the water tank, temperature in the chamber during its steaming was maintained at the level of 20–30 °C. In this period, water vapour absorption by the sublimated (porous) material and its defrosting occurred. The steaming was completed (by closing the influx of steam) at the moment of observation of steam on the defrosted meat (appearance of water droplets on the surface), at the same time determining the steam absorption time by the defrosted sample. Subsequently, the chamber was decompressed and the obtained sample was assessed for the level of its defrosting.

### Assessment indicators in thawing process

The sublimation dehydration level (S_s_) was determined using the Eq. (3), as the relationship between the weight of sublimated ice (m_i_) and the initial sample weight (m_o_), and the weight of the sublimated ice results from the difference between initial sample weight and after the dehydration stage (m_s_):

3 $$ S_{s}  = \frac{{m_{i} }}{{m_{o} }} = \frac{{m_{o}  - m_{s} }}{{m_{o} }} \cdot 100\%  $$

The defrosting level (S_t_) was done by identifying the defrosted field (temperature above 0 °C), non-defrosted field (temperature below 0 °C) and temperature inside the sample (reading from thermocouple). To this end, the defrosted sample was cut centrally, obtaining two measurement samples with thickness of approx. 10 mm. The measurement of the area of the defrosted and non-defrosted surface was done using two methods. Using a thermovision camera (FLIR typ 559384, producent FLIR System Inc. Wilsonville), determining the temperature distribution on the sample cross-section after the thawing process, and on the basis of the sample hardness evaluation, separating the boundaries of the frozen zone from the defrosted zone. The second test was performed by puncturing the meat sample with a needle, separating hard—frozen—from soft—defrosted fields. Then, a series of digital photos of the prepared samples were taken, which were subjected to image analysis in MatLAB environment. The analysis needed additional identification of the boundaries the marked field (the outline was made by joining the puncture sites). The MatLAB algorithm, at the basis of the appointed edges, allows to determine the size of the surface area taking into account its irregular shape. These values were used to calculate the frozen area. Measurement results were used to calculate the degree of defrosting S_t_ according to Eq. (4).

4 $$ S_{t} = \left( {\frac{{F_{t} }}{{F_{c} }} + \frac{{F_{f} }}{{F_{c} }} \cdot \frac{\Delta T}{{\Delta T_{cr} }}} \right) \cdot 100\% . $$

The above equation takes into account the defrosted field (F_t_) in relation to the total cross-section surface area (F_c_) and temperature increment level (ΔT) in relation to the observed range of cryoscopic temperature (ΔT_cr_) relative to the frozen field (F_f_). A more extensive description of the identification of ΔT and ΔT_cr_ is presented in the discussion of results regarding the kinetics of thawing (Fig. 4).

Quality of sample after thawing was assessed using the indicator characterizing the level of initial weight recovery—level of rehydration (S_r_), determined using equation no. 5, as a relationship of the weight after completion of the defrosting process (after rehydration) (m_e_), in regards to the initial sample weight (m_o_).

5 $${S}_{r}= \frac{{m}_{e}}{{m}_{o}}\cdot  100\%$$

Measurement uncertainty Δ for the values: sublimation dehydration level (S_s_), defrosting level (S_t_) and rehydration level (S_r_) were based on the interval estimation theory with the use of Student t-test for the confidence interval α = 0.05, according to equation no. 6.

6 $$ \Delta = \pm \left( {t_{\alpha ,n - 1} \cdot \frac{{\sigma_{n - 1} }}{\sqrt n }} \right) $$

### Statistical analysis

The study was conducted in three replications for each sublimation dehydration variant *S*_*s*_. The obtained results of mass measurement were averaged and listed on graphs as relationships between the level of thawing (S_t_) and level of rehydration (S_r_) on the level of sublimation (S_s_). For the obtained results, standard deviations of dispersion were determined towards the obtained mean values. A HSD Tukey post hoc test was used to check the significance of the influence of sublimation dehydration on level of thawing and sample weight after thawing. Additional statistical analyses were conducted to determine the nature of the level of dehydration on the level of thawing S_t_ = f(S_s_) and sample weight after thawing S_r_ = f(S_s_) by determining the regression functions for this purpose. Each statistical test was carried out in the Statistica 13.1 program.

## Study results

### Results of sample weight change during thawing

**Figure 2:**
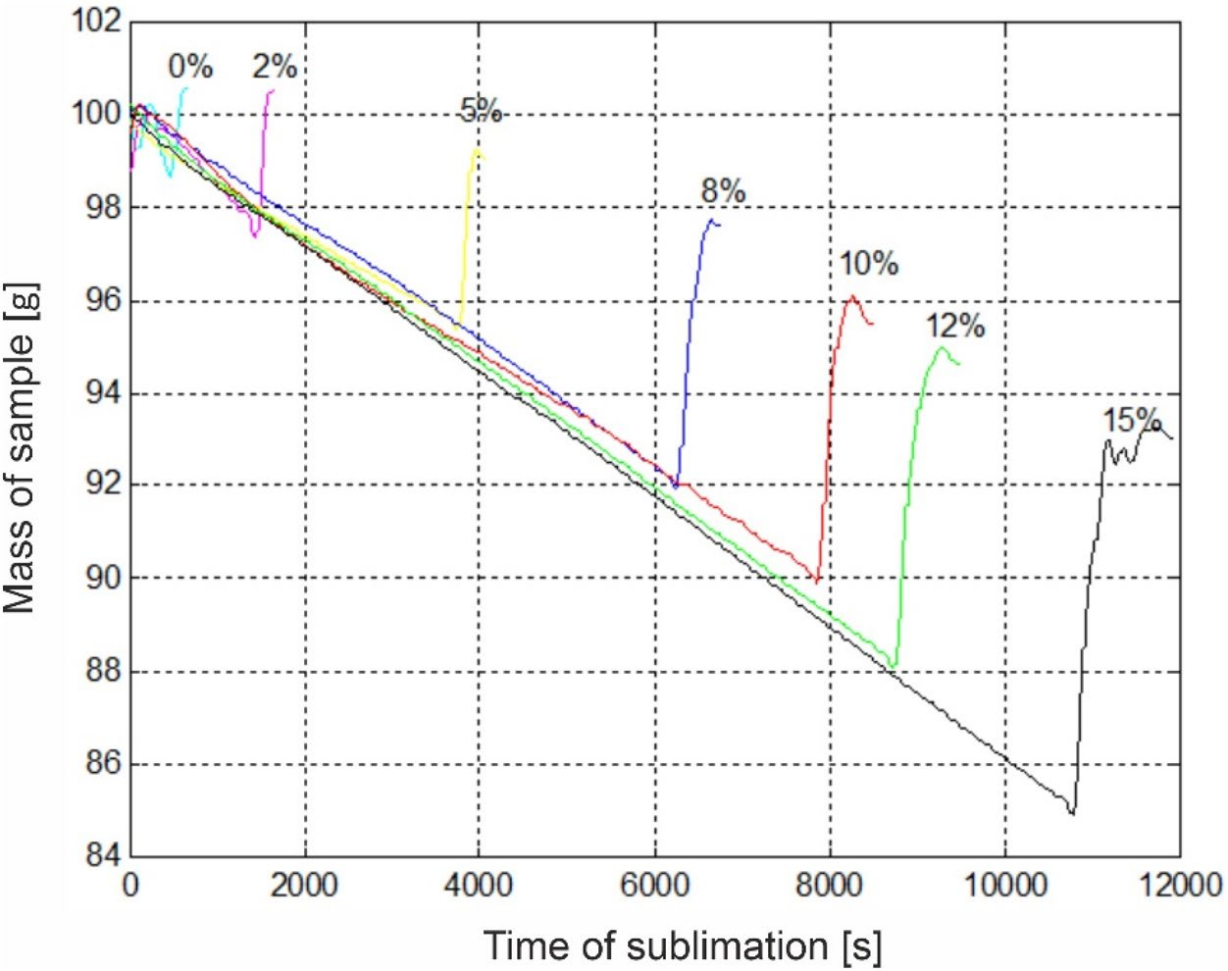
The mass of sample changes during sublimation for different degrees of sublimation dehydration S_s_.

An example of the recorded changes of sample weight during their sublimation for individual dehydration levels is presented in Fig. 2. The course of the curves shall be interpreted in the following manner. The observed linear weight loss corresponds to the stage of preliminary dehydration of meat sample to the level assumed in the experimental plan. On the other hand, the rapid growth increase corresponds to the chamber steaming stage.

For each variant of assumed dehydration level, the final meat sample weight was determined, which constitute the criterion for the completion of the sublimation stage. For dehydration of S_s_ = 8%; 10% and 12% a minor repeated weight loss was observed. This may be caused by too low volume of the porous layer created at the sublimation stage. Condensing water vapour and the drip loss generated during thawing are not fully absorbed by the dehydrated tissue and as the thawing process proceeds, they escape to the outside of the sample, causing weight loss.

### Results of sample temperature change during sublimation and steaming

**Figure 3:**
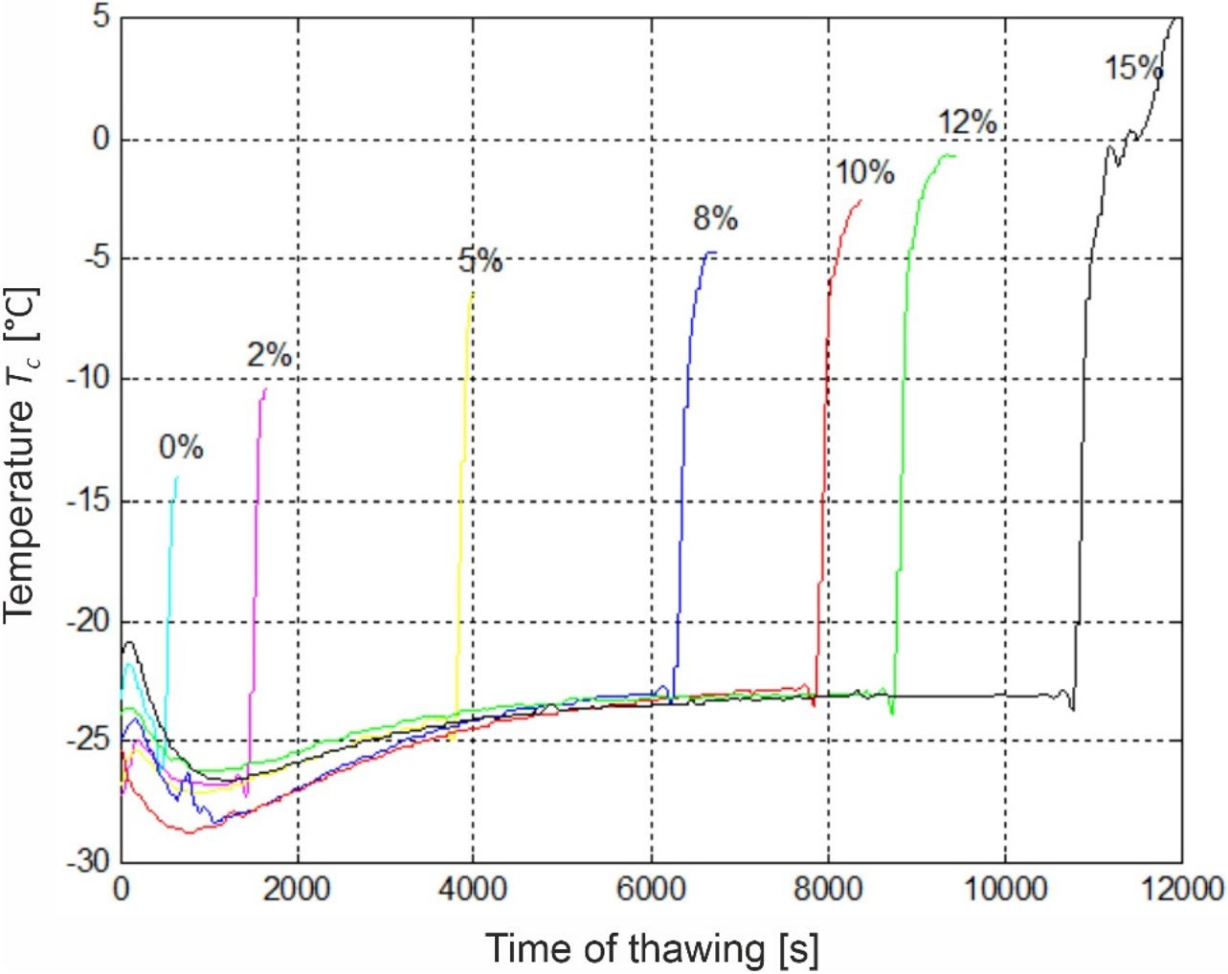
The temperature T_c_ changes in the centre of sample during thawing SRVST method for different degrees of sublimation dehydration S_s_.

Figure 3 presents an example graph of the kinetics of temperature change of meat sample at its geometric center for different *S*_*s*_ sublimation level variants in time.

At the sublimation stage, T_c_ temperature for each of the analyzed variants was maintained in the range between − 28 and − 22 °C. The moment of temperature decrease at the first sublimation stage stems from the repeated freezing of samples. After this stage, temperature of each of the analyzed dehydration variants ranged between − 28 and − 26 °C. The thermal energy necessary to perform sublimation was provided using IR lamps, which after the period of approx. 1000 s resulted in a minor temperature increase of the defrosted sample, followed by its stabilization at the level of approx. T_c_ = − 23 °C. This is particularly visible for the dehydration variants *S*_*S*_ = 5–15%.

**Figure 4:**
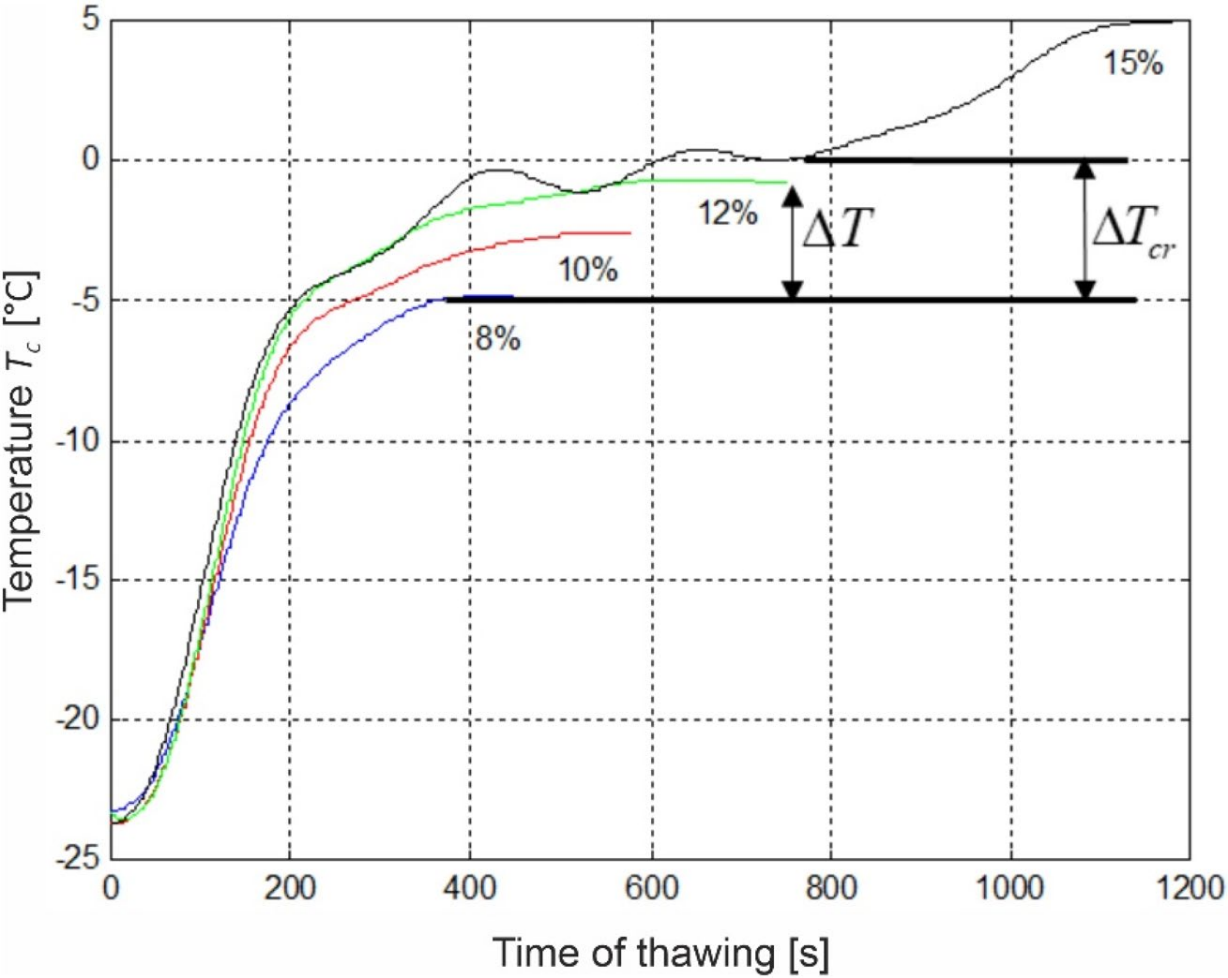
Comparative combination of temperature T_c_ changes at the centre of a sample at the water vapour deposition stage, for 8%, 10%, 12%, and 15% of sublimation dehydration: ΔT the range of phase change occurring within meat during its defrosting for individual sublimation variants; ΔT_cr_ the range of temperatures assumed as cryoscopic temperatures (from − 5 to 0 °C).

Figure 4 presents kinetics of changes of the temperature of defrosted meat sample at the stage of vacuum chamber steaming (temperature increase phase developed from Fig. 3). The ΔT symbol was used to determine the range of phase change occurring within meat during its defrosting for individual sublimation variants, whereas the ΔT_cr_ determined the range of temperatures assumed as cryoscopic (from − 5 to 0 °C). The chart presents result for dehydration variants *S*_*S*_ = 8; 10; 12 and 15%, because for the remaining variants (0; 2 and 5%) the obtained temperature values were not within the assumed cryoscopic range. The steaming stage duration was different for each analyzed sublimation dehydration variant, which is visualized with the course of the measurement curve. The end of the line determines the moment of commencement of water drip from sample surface. The presented temperature change curves show that for the dehydration level of *S*_*S*_ = 12% the sample was completely defrosted at the moment of drip appearance. It assumes internal temperature of approx. − 1 °C. For lower dehydration levels (8 and 10%) remains frozen inside, with temperatures below—4 °C. In turn, for *S*_*S*_ = 15% the sample defrosts before complete rehydration of the dehydrated structure occurs and at final moment of the process the temperature reaches positive values, *T*_*c*_ = 5 °C. The presented kinetics also indicate the defrosting rate. The time in which meat at its geometric center reaches the temperature of 0 °C, is the shorter the greater is the level of ice crystals sublimation.

### Measurement results of temperature field and sample hardness after thawing

**Figure 5:**
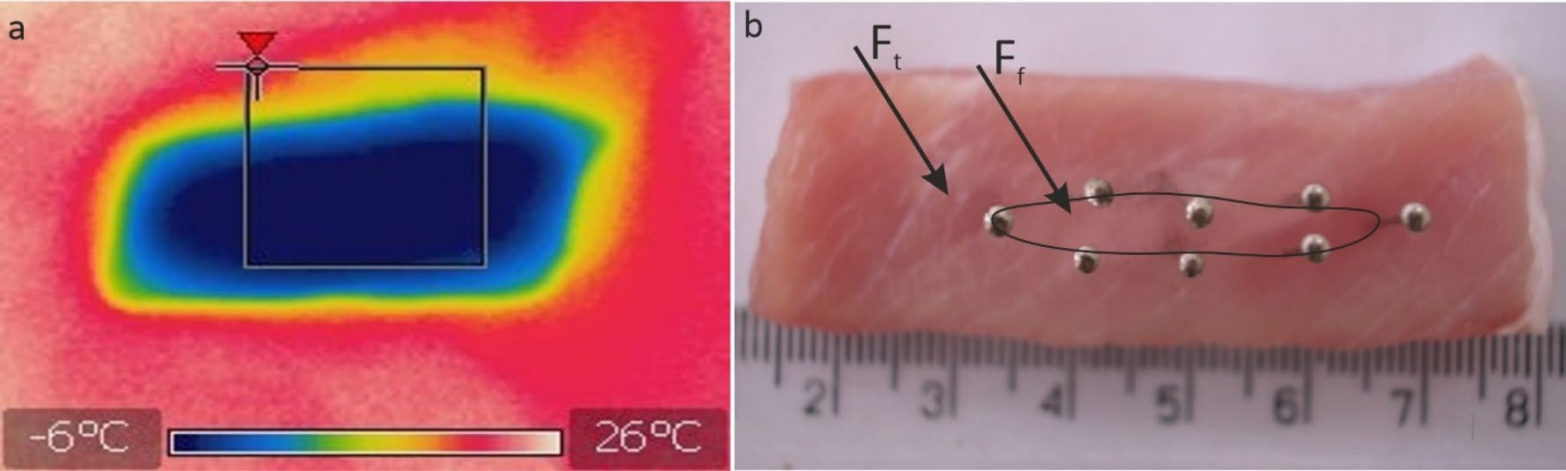
Section through a defrosted sample: (**a**) image of the temperature field from the thermal imaging camera (Type 559384, FLIR Systems. Inc., Wilsonville), (**b**) boundary of the frozen area—identified by puncturing with pins: F_f_—frozen surface, F_t_—thawed surface.

Figure 5 presents examples of photos from the measurements of the defrosted and non-defrosted surface areas of the analyzed sample on its cross-section—using the thermal imaging method 5a and the puncture method 5b.

The result of the measurements was the determination of the size of the defrosted surface (F_t_) in relation to the total cross-sectional area (F_c_) and the size of the frozen surface (F_f_). The thermovision method turned out to be insufficient and it introduced a measurement error resulting from the rapid temperature variability of the sample surface during observation. Thus, further analysis results were obtained based on the results of the puncture method, which turned out to be more unambiguous.

### Results of thawing degree and rehydration degree calculations

**Figure 6:**
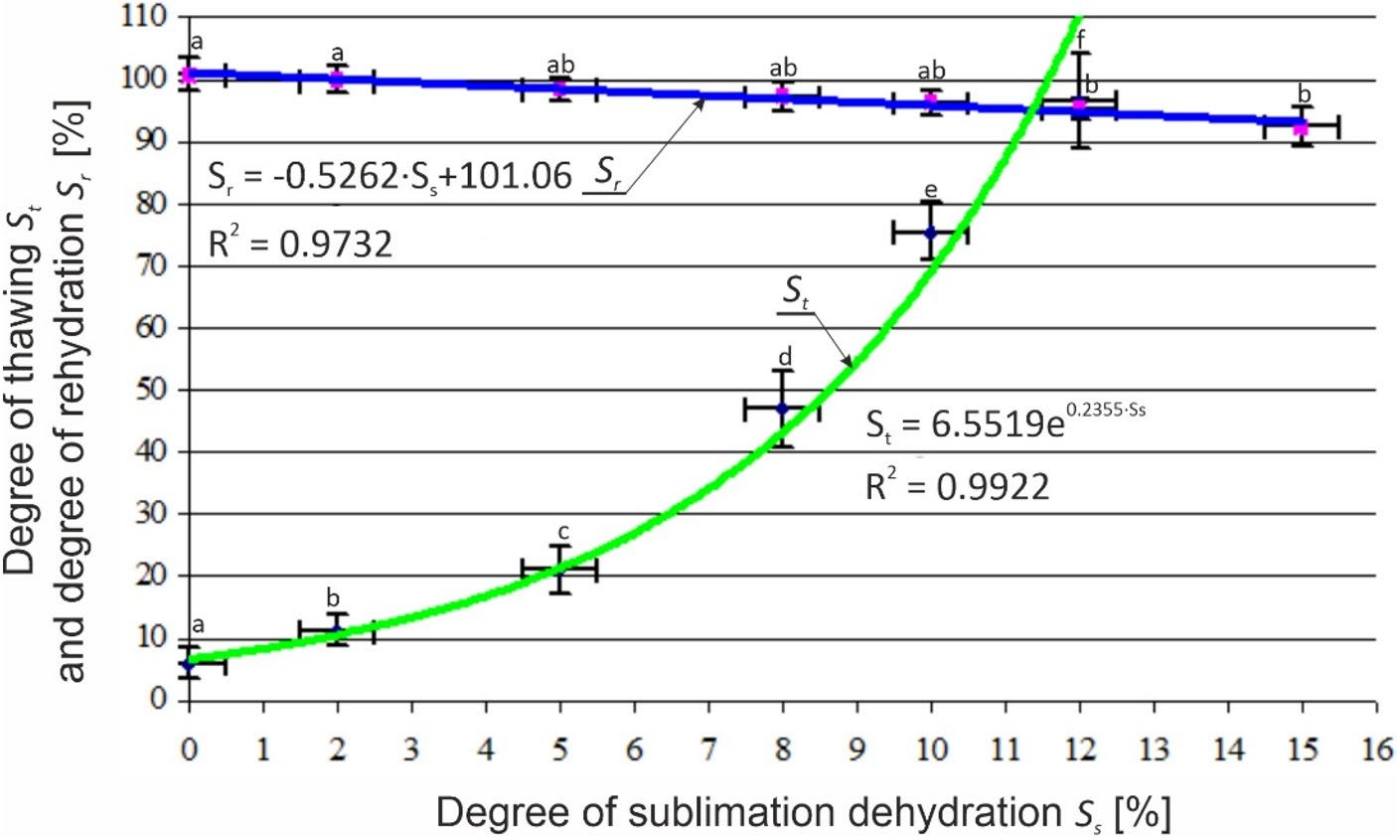
Dependence of the thawing degree S_t_ and the rehydration degree S_r_ for different degrees of sublimation dehydration S_s_.

Figure 6 presents results of measurements and calculations of thawing level (S_t_) and level of primary weight recovery—rehydration level (S_r_) for all tested dehydration variants.

It was observed that with increased sublimation dehydration the level of sample defrosting increases. This relationship (within the tested variable range) can be explained with an exponential function. The obtained results further indicate that with the increase of sublimation dehydration level the level of rehydration is reduced in a linear fashion. In the case of S_s_ = 15% dehydration approx. 8% product weight loss is observed. However, it should be emphasized that this value, according to literature, lies within the normal range for the process of freezing and thawing^31^.

## Discussion

For the assumed variants (with the exception of the 0% zero sample) a sublimation stage characterized by a decrease of weight of the tested samples is visible. At the moment the chamber is steamed, the water vapour diffuses inside the system and gives up heat. As a result of this, the sample weight increases—rehydration occurs. At the moment when steam condensation was observed on the material surface (drip occurred and the sample weight started to reduce), the steaming stage was interrupted. Temperature changes within the sample centers for these two stages of defrosting are presented in Figs. 3 and 4. Chamber steaming resulted in the sample defrosting as a result of heat transfer by the condensing steam on the surface, but primarily within the porous structure of the defrosted material. The condensed steam filled the free spaces of the porous structure of the dehydrated meat sample, and then it condenses inside, giving up latent heat and resulting in its defrosting. Following the rehydration of the dehydrated porous structure, which occurs at a faster rate than the complete sample defrosting, the defrosting process occurs by means of conductivity. The study results are compliant with reports of other authors and indicate that through the vacuum sublimation process—rehydration thawing, also the defrosting rate is largely improved in comparison with the traditional method of vacuum-steam defrosting (zero sample S_s_ = 0%)^11^. This is caused by the latent heat released by the steam condensing within the meat sample. Moreover, it was observed that efficiency of this process also depends on the degree of ice crystal sublimation in the frozen product. The degree of sublimation affects the number of channels (spaces) being formed in the material necessary for the migration of water particles at the steaming stage. The greater the number of the channels (pores), the lower will be the resistance of the exchange of heat and steam weight migrating from the outside to the inside of the material. Thus, not only the rate of thawing will be increased, but also the rehydration time will be prolonged (example variant S_s_ = 15%) and the weight losses will be reduced. Thus, this confirms the hypothesis made on the possibility of vacuum-steam thawing of meat using the preliminary phase of sublimation dehydration.

The analysis of the heat balance of vacuum-steam thawing of meat shows that it is enough to sublimate the mass of ice from the sample, constituting 9% of its mass, in order to, by replenishing this loss with diffusing water vapor, defrost completely the ice remaining in the meat^21,22^. The obtained experimental study results generally confirmed this hypothesis, because S_S_ = 12% dehydration enabled obtaining meat defrosting at the level of 96 ± 0.5%. The main cause for the obtained difference (at the level of 3%) may be creation of a heterogeneous porous structure throughout the entire volume of the defrosted meat sample at the sublimation stage. For the above sublimation variant the rehydration level had a similar value of 96 ± 2%. In defrosted meat (S_s_ = 12% variant) no additional defrosting drip loss or discolorations could be observed, the meat was mildly moist on the surface, did not exhibit drip and it was characterized by natural elasticity. Lower dehydration level (S_S_ < 12%) did not result in complete meat sample defrosting. The amount of ice sublimated in this variant was insufficient to create the appropriate amount of channels and spaces, which resulted in reduced volume of steam penetrating to the frozen product as well as emission of condensation heat. However, dehydration at the level of S_S_ = 15% turned out to be too high. The number of channels (pores) produced for this dehydration variant caused the diffusion of a large volume of water vapor into the thawed sample, and thus a quick thawing process from the inside and overheating of the material. For this sample, the moment of starting water dripping from the surface of the sample (an observable criterion for the end of the thawing process) corresponded to the temperature exceeding 0 °C (the temperature even reached 5 °C), so significantly higher than necessary.

## Conclusions

The study presents a modification of the vacuum-steam thawing method for meat by means of introducing an additional sublimation dehydration stage prior to steaming of the vacuum chamber (SRVST sublimation-rehydration vacuum steam thawing). The selected test subject was the longest muscle of a fattening pig dorsum, from the lumbar part (*m. longissimus lumborum*), which was comminuted by cutting the muscle fibers cross-wise and thus obtaining cooking elements with thickness of approximately 20 mm and weight 100 g ± 5 g. The material was frozen at a temperature − 30 °C and after two weeks it was thawed using the suggested SRVST defrosting method. The method was verified experimentally and based on the obtained study results the following conclusions were drawn.

With regard to the known vacuum-steam defrosting method, in which the heat needed for defrosting comes only from the condensation of water vapor on the surface of the material and then defrosting occurs as a result of its conduction into the material, the method expanded with the stage of preliminary sublimation dehydration is preferable. Defrosting by this method takes place evenly in the entire volume of the material (not from the surface) and at a higher rate. The unfavorable phenomenon of defrosting drip loss is not observed when thawing with this method.

An optimum level of sublimation dehydration exists, enabling defrosting. If the degree of dehydration is too low, the amount of channeling (pores) in the material is too small for the migration of water molecules, resulting in a reduction in the diffusible water vapor volume and thus less condensation heat released. In such a case the thawing rate decreases. However, if the degree of dehydration is too high, there is a risk of overheating of the sample as a result of uncontrolled temperature rise inside the thawed material.

The conducted regression analysis enabled determining the relationship of the effects of the level of dehydration on thawing level S_t_ = f(S_s_) and sample weight after thawing S_r_ = f(S_s_). The developed relationships (linear and exponential) fit well with empirical data. The values of the determination indices for the determined equations are high (R^2^ = 0.97 − 0.99), which allows viewing the equations as a mathematical description of these relationships. Further analyses in this field could be based on the use of the developed equations to model the process (e.g. numerical modelling).

Further research on the development of this method of defrosting is valid, taking into account, inter alia, process parameters, i.e. pressure in the chamber, water vapour volume or process control. Application of this thawing method should also be tested with regards to other products, i.e. fruit and vegetables, or fish blocks, taking into account their dimensions. It is also necessary to conduct further research to determine the process parameters in commercial conditions.
